# Impact of mass testing during an epidemic rebound of SARS-CoV-2: a modelling study using the example of France

**DOI:** 10.2807/1560-7917.ES.2020.26.1.2001978

**Published:** 2021-01-07

**Authors:** Paolo Bosetti, Cécile Tran Kiem, Yazdan Yazdanpanah, Arnaud Fontanet, Bruno Lina, Vittoria Colizza, Simon Cauchemez

**Affiliations:** 1 Mathematical Modelling of Infectious Diseases Unit, Institut Pasteur, UMR2000, CNRS, Paris, France; 2 Collège Doctoral, Sorbonne Université, Paris, France; 3 Infections Antimicrobials Modelling Evolution (IAME) UMR 1137, University of Paris, Paris, France; 4 Emerging Diseases Epidemiology Unit, Institut Pasteur, Paris, France; 5 PACRI Unit, Conservatoire National des Arts et Métiers, Paris, France; 6 National Reference Center for Respiratory Viruses, Department of Virology, Infective Agents Institute, North Hospital Network, Lyon, France; 7 Virpath Laboratory, International Center of Research in Infectiology, INSERM U1111, CNRS—UMR 5308, École Normale Supérieure de Lyon, Université Claude Bernard Lyon, Lyon University, Lyon, France; 8 INSERM, Sorbonne Université, Pierre Louis Institute of Epidemiology and Public Health, Paris, France

**Keywords:** SARS-CoV-2, mass testing, Covid-19, epidemic

## Abstract

We used a mathematical model to evaluate the impact of mass testing in the control of severe acute respiratory syndrome coronavirus 2 (SARS-CoV-2). Under optimistic assumptions, one round of mass testing may reduce daily infections by up to 20–30%. Consequently, very frequent testing would be required to control a quickly growing epidemic if other control measures were to be relaxed. Mass testing is most relevant when epidemic growth remains limited through a combination of interventions.

In autumn 2020, several European countries facing a large increase in coronavirus disease (COVID-19) cases moved back into lockdown. While test–trace–isolate approach remains for now the most efficient way to control an epidemic rebound at the end of these lockdowns, there is debate about optimal ways to use testing [1]. Here, using a mathematical model, we assess the possible impact of mass testing campaigns for severe acute respiratory syndrome coronavirus 2 (SARS-CoV-2) in a scenario of epidemic rebound in Metropolitan France.

## Mass testing using rapid antigen tests

So far, testing for SARS-CoV-2 has mostly targeted symptomatic individuals and contacts of cases. The increasing availability of diverse diagnostic tests now makes it possible to consider a strategy of mass testing, i.e. testing a large proportion of the population in a single campaign to identify and isolate as many infected individuals as possible. The development of rapid antigen tests facilitates the implementation of such an approach since these tests can provide results in less than 30 min compared with 1–2 days for the standard PCR. Although these antigen tests have a lower sensitivity than the PCR test for the diagnostics of SARS-CoV-2, the most sensitive rapid antigen tests have a sensitivity threshold that is sufficient to identify a large proportion of infectious individuals with high viral shedding, ranging from 75 to 97% depending on the test [2,3]. However, even with antigen tests, the implementation of mass testing will be challenging, with an impact that is still to be determined.

## Modelling the spread of SARS-CoV-2 and consecutive campaigns of mass testing

We used a compartmental SEIIR model to describe the spread of SARS-CoV-2 in Metropolitan France [4]. After infection, individuals move between the following compartments: compartment E1, where they have been exposed but are not yet infectious (average duration: 4 days); compartment E2, where they are infectious (i.e. can transmit) but have no symptoms (average duration: 1 day); compartment I, where they are infectious and may be symptomatic (average duration: 3 days); compartment R, where they have recovered. This description leads to a generation time of ca 7 days [5], consistent with existing data on chains of transmission and viral excretion [6,7].

In our baseline scenario, we deliberately considered optimistic assumptions to derive an upper bound of the impact of mass testing. We assumed that an infectious individual (i.e. in compartments E2 or I) has a probability of testing positive with an antigenic test equal to the sensitivity Se = 90% [2,3] and that a person testing positive will, thanks to self-isolation, reduce onward transmissions by *ρ* = 70% on average. In sensitivity analyses, we also considered Se = 60% or 75% and *ρ* = 50% or 90%.

For the sake of exercise, we assume that France experiences an epidemic rebound starting on 4 January 2020. Assuming a basic reproduction number *R_0_* = 0.9 from 30 November 2020, the start of the second French lockdown, to 4 January 2021, we expect ca 8,000 infections daily, with about half of them detected, and 13% of the population of Metropolitan France being infected from January 2020 until 4 January 2021. From 4 January 2021, we consider rebound scenarios where the number of daily infections doubles every 10 (effective reproduction number (*R_e_*) = 1.6), 14 (*R_e_* = 1.4), 17 (*R_e_* = 1.3) and 21 (*R_e_* = 1.2) days. As a reference, *R_e_* was estimated at 1.4 from hospitalisation data in mid-October in Metropolitan France [8].

We assume that the first testing campaign starts on 4 January and is repeated every 1–30 days, with 0–90% of the population tested during each campaign. We assume that mass testing is performed in a single day. We assess the impact of these campaigns until 1 May 2021.

### Impact of mass testing on epidemic dynamics

**Figure 1:**
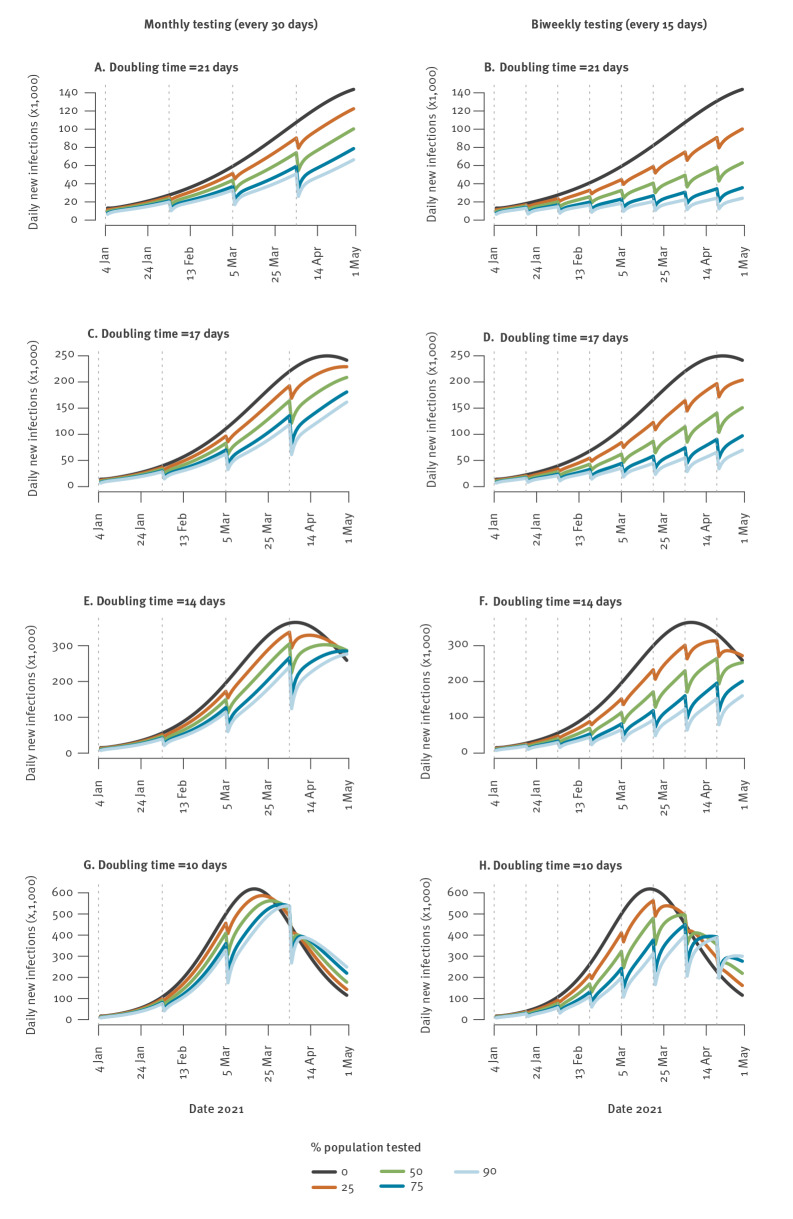
Expected number of daily SARS-CoV-2 infections with monthly or biweekly testing campaigns, by date and percentage of population tested, France, 4 January–1 May 2021

Figure 1 shows epidemic dynamics for monthly and biweekly campaigns of mass testing starting on 4 January 2021. For an epidemic rebound with a doubling time of 21 days, we would expect 140,000 infections per day by 1 May in the absence of mass testing. A monthly campaign of mass testing in which 75% of the population is tested would reduce that number to 80,000 infections per day, and a biweekly campaign to 35,000. For a doubling time of 14 days, a monthly campaign testing 75% of the population would reduce daily infections at the peak from 360,000 to 285,000, and biweekly campaigns would further reduce daily infections to 200,000.

SARS-CoV-2: severe acute respiratory syndrome coronavirus 2.

Panels A, C, E and G (left column): monthly campaigns.

Panels B, D, F and H (right column): biweekly campaigns.

The figure presents epidemiological scenarios where the number of infections doubles every 21 days (panels A and B), 17 days (panels C and D), 14 days (panels E and F) and 10 days (panels G and H). We assumed that the probability to detect an infectious individual with an antigenic test is Se = 90% and the reduction in onward transmission following a positive test is *ρ* = 70%.

**Figure 2:**
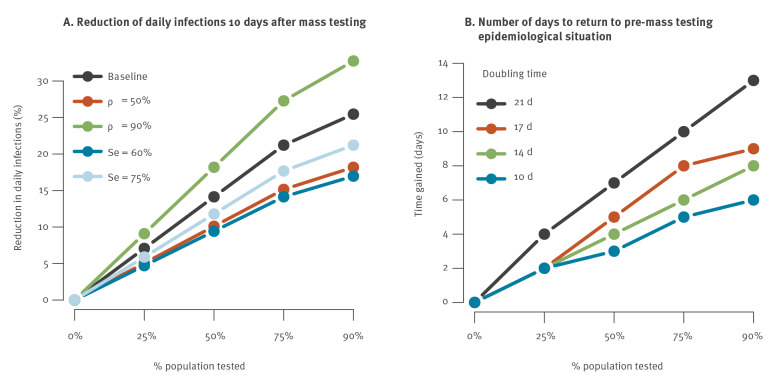
Impact of a single mass testing campaign for SARS-CoV-2 on (A) reduction of daily infections 10 days after mass testing and (B) number of days to return to pre-mass testing epidemiological situation, France, 4 January–1 May 2021

In our baseline scenario, a single campaign targeting 75% of the population reduced the number of daily infections that occur 10 days after the campaign by 21% (Figure 2A). We obtained 14% and 18% reduction, respectively, for a sensitivity Se of 60% and 75%, and 15% and 27% reduction for an effectiveness of self-isolation *ρ* of respectively 50% and 90%. Results are insensitive to the value of the *R_e_* (data not shown). These reductions may have limited impact on the overall dynamics in a context of quick epidemic rebound. For example, in our baseline scenario, if the number of infections doubles every 21 days and 75% of the population is tested, we expect that it would take 10 days to get back to the epidemiological situation observed before mass testing (Figure 2B). For a doubling time of 14 days, we would only gain 6 days.

d: days; SARS-CoV-2: severe acute respiratory syndrome coronavirus 2.

Panel A shows the percentage reduction in the number of daily infections occurring 10 days after the campaign, for different scenarios. In our baseline scenario, the probability to detect an infectious individual with an antigenic test is Se = 90% and the reduction in onward transmission following a positive test is *ρ* = 70%. We consider alternative scenarios with *ρ* = 50% or 90% and Se = 60% or 75%.

Panel B displays the number of days it takes for the number of daily infections to return to the levels observed before mass testing, as a function of the doubling time in the baseline scenario.

**Figure 3:**
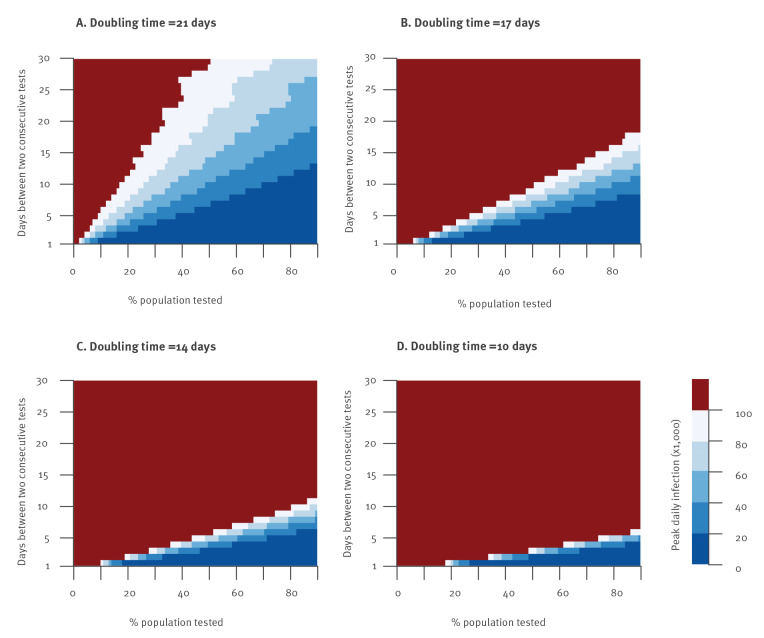
Expected maximum number of daily SARS-CoV-2 infections as a function of the number of days between consecutive campaigns and the proportion of the population tested in each campaign, for different doubling times, France, 4 January–1 May 2021

Figure 3 shows the maximum daily number of infections observed from 4 January to 1 May as a function of the number of days between consecutive campaigns and the proportion of the population tested in each campaign. In our baseline scenario, for a doubling time of 21 days, to ensure that the daily number of infections remains below 80,000, 60,000 and 40,000 up to 1 May, a campaign testing 75% of the population would need to occur every 30, 22 and 16 days, respectively. For a doubling time of 14 days, testing of 75% of the population should occur every 6 days to remain below 40,000 daily infections.

SARS-CoV-2: severe acute respiratory syndrome coronavirus 2.

We assumed that the probability to detect an infectious individual with an antigenic test is Se = 90% and the reduction in onward transmission following a positive test is *ρ* = 70%.

### Sensitivity analysis

**Figure 4:**
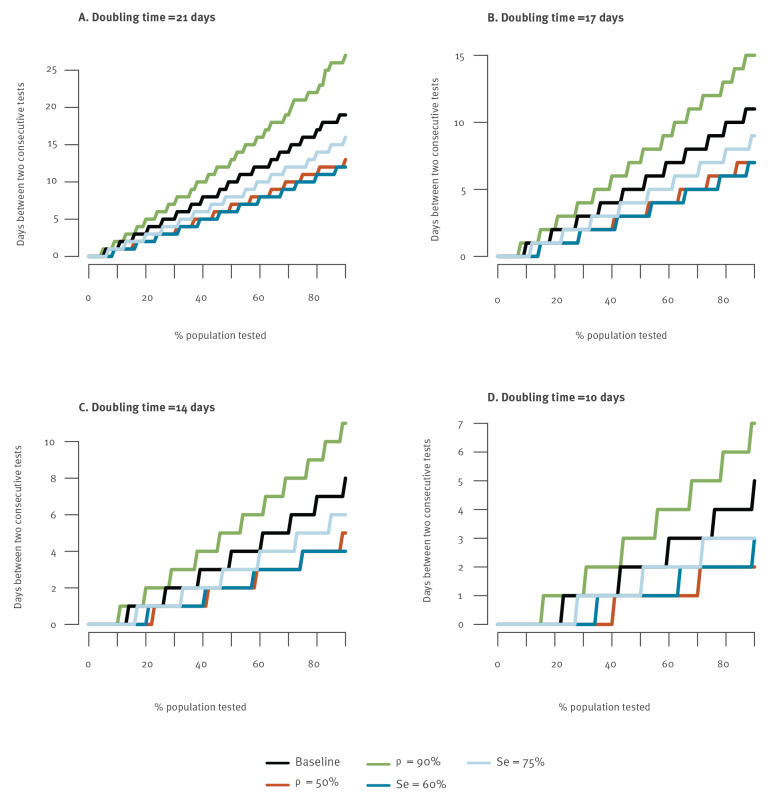
Frequency of mass testing campaigns necessary to keep the number of daily SARS-CoV-2 infections below 40,000, as a function of the proportion of the population tested in each campaign, for different modelling assumptions, France, 4 January–1 May 2021

We explored the sensitivity of our results to model assumptions (Figure 4). To observe fewer than 40,000 daily infections from 4 January to 1 May with a doubling time of 21 days, testing of 75% of the population needs to be repeated every 21 and 11 days, respectively, when the effectiveness of self-isolation following a positive test is *ρ* = 90% and 50%. Such campaigns need to be repeated every 12 and 10 days for a sensitivity Se of the test of 75% and 60%, respectively.

SARS-CoV-2: severe acute respiratory syndrome coronavirus 2.

In our baseline scenario, we assumed that the probability to detect an infectious individual with an antigenic test is Se = 90% and the reduction in onward transmission following a positive test is *ρ* = 70%. We considered alternative scenarios where the reduction in transmission is *ρ* = 50% and 90% and where the probability to detect an infectious individual is Se = 60% and 75%.

## Discussion

We used a simple mathematical model to highlight the potential and limits of mass testing for the control of SARS-CoV-2 epidemics. Under optimistic assumptions, we find that one round of mass testing may reduce daily infections by up to 20–30%. Consequently, very frequent testing would be required to control a quickly growing epidemic if other control measures were to be relaxed. Mass testing is therefore most relevant when epidemic growth remains limited thanks to a combination of interventions. These results are consistent with another modelling study from the Netherlands, which concluded that regular universal screening alone may not allow for re-opening of society [9].

In combination with interventions that are able to slow down epidemic growth, high frequency of screening remains an important additional contribution to epidemic control [7]. However, the logistics, effectiveness of self-isolation and voluntary participation of the population in repeated mass testing campaigns need to be assessed carefully. So far, few pilot studies have been conducted in geographically confined areas and/or populations of comparatively limited size and one-time approaches have required several days of implementation [10-12]. Limited compliance with isolation measures in those with a positive test would largely compromise such efforts. Here we considered an optimistic scenario, with 70% reduction of onward transmission following testing (with values ranging from 50–90%), but survey data from the United Kingdom point to smaller compliance rates of ca 10% [13].

During a mass testing campaign in Slovakia that was implemented in multiple rounds, the prevalence of infection dropped by ca 60% in the week between the first and second round of mass testing [11]. The campaign happened alongside important restrictions, including a 1-week lockdown. This makes it difficult to dissociate the impact of mass testing from that of other interventions. For example, if *R_e_* was 0.6–0.7 as a consequence of other measures, we would expect the prevalence of infection to drop by ca 30–40% per week in the absence of mass testing. In such a scenario, mass testing could have contributed to the additional 20–30% reduction, which would be roughly consistent with estimates under our most optimistic scenarios. The strategies considered in our assessment required isolation only of positive cases. In contrast, in Slovakia, the whole household was quarantined when a case was detected, which is likely to have a larger impact on spread.

An important limitation of mass testing is that, when the campaign begins, approximately half of the individuals who are infected are still in the latent phase E1 so they do not shed sufficient virus to test positive. These individuals will not be detected and will become infectious after the campaign, again fuelling the epidemic. This problem can be partly mitigated by extending isolation measures to include household contacts, as in Slovakia, and performing robust contact tracing [14]. For a given number of tests, impact might be higher if the tests target areas, populations or places of higher incidence.

Our study has limitations. Firstly, it relied on a deterministic model that may imperfectly capture epidemic dynamics when the daily number of infections is low. In such situations, it may take longer for the epidemic to rebound than anticipated by the model. Secondly, we do not address the logistical challenges involved in regular mass testing and assume that individuals are tested in a single day. In practice, such a campaign is likely to occur over multiple days, though we do not expect that this would radically modify our results.

## Conclusions

Mass testing may help reduce the daily number of SARS-CoV-2 infections, but campaigns may need to be implemented very frequently to control a quickly growing epidemic. As a result, mass testing is most relevant when epidemic growth remains limited by a combination of interventions.
